# PLGA particulate subunit tuberculosis vaccines promote humoral and Th17 responses but do not enhance control of *Mycobacterium tuberculosis* infection

**DOI:** 10.1371/journal.pone.0194620

**Published:** 2018-03-19

**Authors:** Anneliese S. Ashhurst, Thaigarajan Parumasivam, John Gar Yan Chan, Leon C. W. Lin, Manuela Flórido, Nicholas P. West, Hak-Kim Chan, Warwick J. Britton

**Affiliations:** 1 Tuberculosis Research Program, Centenary Institute, University of Sydney, Camperdown, New South Wales, Australia; 2 Faculty of Pharmacy, University of Sydney, Sydney, New South Wales, Australia; 3 School of Chemistry and Molecular Biosciences and Australian Infectious Disease Research Centre, University of Queensland, Brisbane, Queensland, Australia; 4 Discipline of Medicine, Sydney Medical School, University of Sydney, Sydney, New South Wales, Australia; Fundació Institut d’Investigació en Ciències de la Salut Germans Trias i Pujol, Universitat Autònoma de Barcelona, SPAIN

## Abstract

Tuberculosis places a staggering burden on human health globally. The new World Health Organisation End-TB Strategy has highlighted the urgent need for more effective TB vaccines to improve control of the disease. Protein-based subunit vaccines offer potential as safe and effective generators of protective immunity, and the use of particulate vaccine formulation and delivery by the pulmonary route may enhance local immunogenicity. In this study, novel particulate subunit vaccines were developed utilising biodegradable poly(lactic-*co*-glycolic acid) (PLGA) slow-release particles as carriers for the *Mycobacterium tuberculosis* lipoprotein MPT83, together with the adjuvants trehalose-dibehenate (TDB) or Monophosphoryl lipid A (MPL). Following delivery by the pulmonary or subcutaneous routes, the immunogenicity and protective efficacy of these vaccines were assessed in a murine model of *M*. *tuberculosis* infection. When delivered peripherally, these vaccines induced modest, antigen-specific Th1 and Th17 responses, but strong anti-MPT83 antibody responses. Mucosal delivery of the PLGA(MPT83) vaccine, with or without TDB, increased antigen-specific Th17 responses in the lungs, however, PLGA-encapsulated vaccines did not provide protection against *M*. *tuberculosis* challenge. By contrast, peripheral delivery of DDA liposomes containing MPT83 and TDB or MPL, stimulated both Th1 and Th17 responses and generated protection against *M*. *tuberculosis* challenge. Therefore, PLGA-formulated vaccines primarily stimulate strong humoral immunity, or Th17 responses if used mucosally, and may be a suitable carrier for vaccines against extracellular pathogens. This study emphasises the critical nature of the vaccine carrier, adjuvant and route of delivery for optimising vaccine efficacy against TB.

## Introduction

Despite considerable research efforts, tuberculosis (TB) remains a staggering burden on global health with 10.4 million new cases and 1.7 million deaths in 2016 [1]. Of the estimated two billion individuals infected, 90% effectively control the infection via the host immune response but do not eliminate it, providing a reservoir for reactivation and subsequent transmission. No new vaccines have been approved for human use since the development of the live attenuated *M*. *bovis* bacille Calmette-Guérin (BCG). BCG has been widely used since 1921, but has highly variable efficacy, does not prevent transmission and also possesses significant safety concerns for immunocompromised individuals [2, 3]. The 2015 World Health Organisation End-TB Strategy identifies the urgent need for more effective and easily administrable vaccines, as the optimum tool for controlling TB. Exploring alternative routes of vaccine delivery, antigens and adjuvant formulations may aid this development.

There is growing interest in pulmonary vaccine delivery, which eliminates the use of needles and follows the natural route of infection with *M*. *tuberculosis*. The large surface area of the lungs exposes substantial regions of lymphoid tissue to antigen and adjuvant, which may provide an opportunity to generate protective resident memory T-lymphocytes at the site of exposure [4, 5]. This may be particularly advantageous in the case of *M*. *tuberculosis* infection, where priming and recruitment of effector T-lymphocytes to the lungs only occurs after one to two weeks, allowing unchecked growth of the organism [6, 7]. Viral vectored TB vaccines have shown potential in animal models [8] however significant safety concerns exist in particular for pulmonary immunisation, and repeat use may be limited due to host immune responses to the vector. Protein-based subunit vaccines offer potential as safe and effective generators of protective immunity more suitable for repeat use, but require effective immunostimulatory adjuvants. There are few adjuvants currently approved for human vaccines, and these are generally poor inducers of the T-helper type 1 (Th1)-based immunity required for protection from *M*. *tuberculosis* [9]. In addition, there are minimal data available on the efficacy of adjuvants for mucosal vaccination, as well as how they may be combined with antigen.

Co-delivery of antigen and adjuvants in a slow release particulate formulation has been suggested to increase immunogenicity and protective efficacy [10, 11]. Numerous platforms exist for this purpose, however the use of poly(lactic-*co*-glycolic acid) (PLGA) particles is gaining attention. PLGA is a copolymer that is biocompatible and biodegradable, breaking down into lactic and glycolic acid in vivo. It has been used in a number of FDA-approved drug delivery and diagnostic strategies as well as in therapeutic devices [12]. PLGA particles possess a matrix-like core ideal for encapsulation and protection, surrounded by a stabilising polymer [13]. Polyvinyl alcohol (PVA) is one of the most commonly used stabilisers and is reported to be non-toxic [14–17]. Previously, Kirby et al (2008) investigated the combination of PLGA particles with a fusion of two commonly investigated *M*. *tuberculosis* vaccine proteins, Ag85B and ESAT6, together with the adjuvants dimethyl dioctadecyl ammoniumbromide (DDA) and trehalose-dibehenate (TDB). When given by subcutaneous injection, this combination was able to generate a modest antigen-specific IFNγ response as well as significant humoral immunity in mice [18]. We wished to examine whether the use of pulmonary delivery, or the incorporation of alternative antigens or adjuvants into PLGA-based particulate vaccines, may enhance Th1 and Th17 immune responses implicated in protection against *M*. *tuberculosis*, and in addition to assess the protective efficacy of these vaccines in a murine challenge model.

Until now only a limited number of *M*. *tuberculosis* antigens have been included in TB vaccines entering clinical trials [19]. Therefore to broaden the range of antigens under study, we selected the secreted protein MPT83 (Rv2873) to include in PLGA particles [20–24]. This glycosylated lipoprotein is recognised by humans with *M*. *tuberculosis* infection, resulting in significant antibody and cellular responses [21]. In murine models, MPT83 DNA-, RNA- and protein-based vaccines stimulated protective immune responses [21, 25], including when MPT83 was ligated to a TLR2 ligand as a dry powder pulmonary vaccine [26]. MPT83 therefore shows potential as a protective vaccine antigen that has an added benefit of not impairing commonly used diagnostic tests designed to establish *M*. *tuberculosis* infection. The adjuvant TDB, a synthetic analogue of the mycobacterial virulence factor trehalose-6,6-dimycolate, has shown potential in preclinical vaccine studies in combination with DDA liposomes [27, 28]. TDB interacts with the surface receptor, Mincle, activating NF-κB by Card9 signalling and the Nlrp3 inflammasome leading to IL-1β secretion [29] and induction of Th1/Th17 responses [30]. A second adjuvant with potential is Monophosphoryl lipid A (MPL), a Toll-like receptor(TLR)-4 ligand comprised of the lipid A portion of lipopolysaccharide (LPS) that has the (R)-3-hydroxytetradecanoyl group and the 1-phosphate removed [31, 32]. MPL stimulates dendritic cells to produce TNFα and IL-12, leading to Th1 responses [33]. MPL has been administered subcutaneously (s.c) and intra-nasally (i.n) in animal models [34, 35] and is approved for use in clinical trials.

In this study therefore, novel biodegradable PLGA particles were used as a carrier for the protective protein antigen, MPT83, and adjuvants targeting the pattern recognition receptors (PRRs), Mincle (TDB) or TLR4 (MPL). These were compared to DDA liposome-based vaccines incorporating the same antigen and adjuvants. The immunogenicity and protective efficacy of these vaccines were assessed in a murine model of *M*. *tuberculosis* infection, including assessment of Th1, Th17 and humoral responses, with a comparison of mucosal and peripheral vaccine delivery.

## Materials and methods

### Bacterial strains and growth conditions

*M*. *bovis* BCG Pasteur 1173P2 and *M*. *tuberculosis* H37Rv (ATCC 27294) were cultured at 37 °C in Middlebrook 7H9 (Difco) broth with albumin-dextrose-catalase (ADC; 10% v/v), Tween-80 (0.05% v/v) and glycerol (0.2% v/v). To enumerate, cultures were plated onto Middlebrook 7H11 (Difco) agar, with oleic-acid-albumin-dextrose catalase (OADC; 10% v/v) and glycerol (0.5% v/v).

### Recombinant MPT83 expression and purification

Recombinant MPT83 was produced as previously described [26]. Briefly, chemically competent *E*. *coli* BL21(DE3) were transformed with the protein expression vector pAT2. After subculture until mid-log phase, MPT83 expression was induced with IPTG (0.5 mM). Bacterial pellets were resuspended in lysis-solubilisation buffer (8 M urea, 50 mM Tris pH 7.5, 300 mM NaCl) and incubated on a rolling circle (4 °C, >4 hrs). After removal of insoluble cell debris by centrifugation, His-tagged MPT83 was purified by Co^2+^ charged, immobilised metal affinity chromatography (IMAC) (Talon, Clontech, CA). MPT83 was dialysed against phosphate buffered saline (PBS), concentrated by centrifugal filtration (Amicon Ultra-15, MWCO 10kDa, Millipore, MA) and 0.22 μm filter sterilised (Millipore). Purity was confirmed by SDS-PAGE and concentration determined using a Pierce BCA Protein Assay Kit (Thermo-Scientific).

### SDS-PAGE

After heating (>98 °C, 10 min) in denaturing sample buffer (60 mM Tris (pH 6.8), 1% w/v SDS, 1% v/v β-mercaptoethanol, 10% v/v glycerol, 0.01% w/v bromophenol blue), samples were separated on a two phase 4%/12% SDS-polyacrylamide stacking/resolving gel alongside a MW standard (Bio-Rad, CA) and visualised by Coomassie blue staining.

### Preparation of adjuvants

MPL (1 mg/ml; Sigma, MO) was prepared in 0.2% triethylamine, with repetitive heating to 70 °C for 30 s and brief sonication. TDB lyophilised powder (Avanti Polar Lipids, AL) was prepared as a suspension. After addition of DMSO and brief agitation, TDB was heated to 60 °C for 30 s, then vortexed to form a suspension (50 mg/ml). The suspension was diluted with endotoxin free dH_2_O to yield TDB at 5 mg/ml, agitated, heated at 60 °C for 15 min to aid dispersion, and homogenised by vortexing for 30 s.

### Production of PLGA particulate vaccines and assessment of protein content

Production and assessment of PLGA particles was adapted from published protocols [10, 11, 36–38]. MPT83 and/or TDB/MPL were encapsulated in PLGA particles utilising a water-in-oil-in-water (w/o/w) double emulsion technique, followed by solvent evaporation, washing and drying. The internal aqueous phase consisted of PBS, MPT83 in PBS, and/or TDB/MPL. The quantity of MPT83 and adjuvants utilised was adjusted depending on the desired final concentration per mg PLGA (10–20 μg MPT83 and 10 μg TDB or 37.5 μg MPL), based on 60–70% encapsulation of MPT83 determined by optimisation experiments. The internal water phase was combined at a 4:5 ratio in a glass test tube with PLGA (60 mg/ml in dichloromethane; 65:25, LACTEL Polymer, Durect Corporation) and probe sonicated (60 s, power 4; Branson Sonifier 450, Branson Ultrasonics, Danbury). The primary water in oil emulsion (w/o) was added to 2% w/v PVA (8.3 x the volume of the w/o) in a 50 ml glass beaker and similarly sonicated, to provide a w/o/w double emulsion. The solution was subjected to solvent evaporation and particle hardening for 4 hr at room temperature (25 °C) by magnetic stirring. The particles were recovered by centrifugation (10,000 *g*, 15 min; Beckman L90-K Ultracentrifuge, Beckman Coulter Australia Pty Ltd.), washed three times with sterile ultra-pure distilled water and resuspended. The washed suspension was dripped into liquid nitrogen to aerate prior to freeze drying (Alpha 2–4 LDplus, Martin Christ, Germany), then stored at 4 °C in desiccant. Particles were approximately 0.5–2 μm, as measured by laser diffraction and scanning electron microscopy, as previously described [17]. The vaccine was resuspended in sterile PBS prior to vaccination of mice (1 mg/dose). To extract protein for quantitation, particles were incubated in 0.1 M NaOH and 5% SDS overnight with agitation. Insoluble debris was pelleted and the supernatant assayed for protein content by BCA. Alternatively, PLGA particles were prepared for SDS-PAGE by heating (>98 °C, 10 min) in denaturing sample buffer, and assayed against a known protein standard. Protein integrity, measured by SDS-PAGE, was maintained following storage as a powder at 4 °C for at least one month.

### Preparation of DDA liposomal vaccines

DDA (5 mg/ml; Sigma) was prepared in sterile distilled water at 80 °C with regular agitation for 20 min. Vaccines were prepared in sterile PBS, with the addition of MPT83 (10 μg), DDA (62.5 μg/i.n dose, 250 μg/s.c dose) and TDB (50 μg/dose) or MPL (25 μg/dose). The components were vortexed then incubated (30 min, RT) to allow protein to adsorb to DDA, and emulsified by vortexing again immediately prior to vaccination.

### Mice

Six to eight week old female C57BL/6 mice were obtained from the Animal Resources Centre (Perth, W.A.) or Animal BioResources (Moss Vale, NSW, Australia). Mice were housed in the Centenary Institute animal facility under SPF conditions. All murine experiments were conducted in strict accordance with the approvals granted by the University of Sydney Animal Ethics Committee (K75/10-2011/3/5565) or the Sydney Local Health District Animal Welfare Committee (2013/054 and 2013/075).

### Immunisation of mice and collection and processing of organs

Mice receiving s.c vaccines were anaesthetised with gaseous isofluorane (4%) and injected at the base of tail with 200 μl. For i.n immunisation, mice anaesthetised by intra-peritoneal injection of ketamine/xylazine (50 mg/6.25 mg/kg) received vaccine in 50 μl isotonic solution applied to the nares, which due to the large volume reached both the nasal mucosa and lungs. Mice received three vaccinations two weeks apart then immunogenicity was assessed at four weeks after the last vaccination. Lungs were perfused with PBS and heparin (20 U/ml; Sigma) and digested with collagenase type 4197 (50 U/ml; Freehold NJ) and DNase I (13 μg/ml; Sigma) in RPMI media (37 °C, 45 min) prior to homogenisation and filtration. The spleen and draining lymph nodes (DLN), the mediastinal lymph node (MLN) of i.n immunised mice or the inguinal lymph nodes (ILN) of s.c immunised mice, were homogenised through a 70 μm sieve. Erythrocytes were removed by ACK lysis buffer. Sera was collected following cardiac puncture.

### Murine IFNγ T-lymphocyte responses

Antigen-specific IFNγ-secreting lymphocytes from mice were enumerated by ELISPOT as previously described [21]. Purified MPT83 (10 μg/ml) was used as recall antigen, with Concanavalin A (ConA) (3 μg/ml) or media alone as controls.

### Polyfunctional T-lymphocyte responses to immunisation

Antigen-specific cytokine production was enumerated by intra-cellular immunostaining and flow cytometry. 4×10^6^ lymphocytes were stimulated for 4 hr with MPT83 (10 μg/ml), or as controls with anti-mouse CD3 (1452C11, 5 μg/ml) and anti-mouse CD28 (37.51, 5 μg/ml; BD Pharmingen) or media alone. Brefeldin A (10 μg/ml; Sigma) was added and further incubated (16 hr) to allow intracellular accumulation of cytokine. Fc receptors were blocked with anti-mouse CD16/CD32 (2.4G2; BD Biosciences) in FACS wash (PBS with 2% FCS). Surface markers were labelled with monoclonal anti-mouse CD8-APCCy7 (53–6.7; BD Pharmingen, San Jose, CA), CD4-PECy7 (RM4-5; BD Pharmingen), CD3-PerCPCy5.5 (17A2; Biolegend, San Diego, CA) and live/dead fixable blue dead cell stain (1:200; Invitrogen, CA). Following washing, cells were fixed using BD Cytofix/perm and washed with BD Perm/Wash. Intracellular cytokines were labelled with monoclonal anti-mouse IFNγ-FITC (XMG1.2; BD Pharmingen), IL-17A-PB (TC11-18H10.1; Biolegend), TNFα-PE/APC (MP6-XT22; Biolegend) and IL-2-APC/PE (JES6-5H4; Biolegend) and washed. The concentration of antibodies used for immunolabelling was determined by in-house titration experiments. All immunostained cells were fixed in 10% neutral buffered formalin prior to acquisition using an LSRFortessa or LSRII 5L flow analyser (BD Biosciences) and analysis of cytokine expression using the FlowJo Boolean gating tool (Tree Star Inc.).

### Cellular proliferation in response to immunisation

2×10^5^ leukocytes from spleens or LN were stimulated for 72 hr with MPT83 (10 μg/ml) or ConA (3 μg/ml), then pulsed with tritiated (^3^H)-thymidine (5 μCi/ml; Perkin Elmer, MA) for 6 hr. Cells were transferred to glass fibre filters (Perkin Elmer) using a plate harvester (Wallac, MA), liquid scintillant added (Betaplate scint, Perkin Elmer) and incorporation measured (Perkin Elmer).

### Murine serum IgG responses

Serum anti-MPT83 IgG titres were determined by ELISA as previously described [26]. Briefly, plates were coated with purified MPT83 (2 μg/ml) and blocked with 3% w/v BSA. Diluted sera were added (1 hr, 37 °C), washed and incubated with alkaline phosphatase-conjugated goat anti-mouse IgG to detect bound IgG (1:2000; Sigma). Substrate was added to develop the assay and the absorbance read at 405 nm (POLARstar Omega, BMG Labtech).

### Experimental *M*. *tuberculosis* infection

Six weeks after the last immunisation, a low-dose aerosol infection (~100 CFU) was delivered to mice in an inhalation exposure system (Glas-Col, Terre Haute, IN) [26]. Four weeks after challenge, serial dilutions of lung and spleen homogenates were plated to enumerate the bacterial loads.

### Statistical analysis

Statistical analysis was performed using GraphPad Prism 6 or 7 software (GraphPad Software, La Jolla, CA). Differences between two groups were analysed by Students *t*-test, or between multiple groups by analysis of variance (ANOVA) with Bonferroni post-hoc comparison, and were considered significant when the *P* values were ≤0.05.

## Results

### T-cell IFNγ responses to mucosal or peripheral immunisation

**Fig 1:**
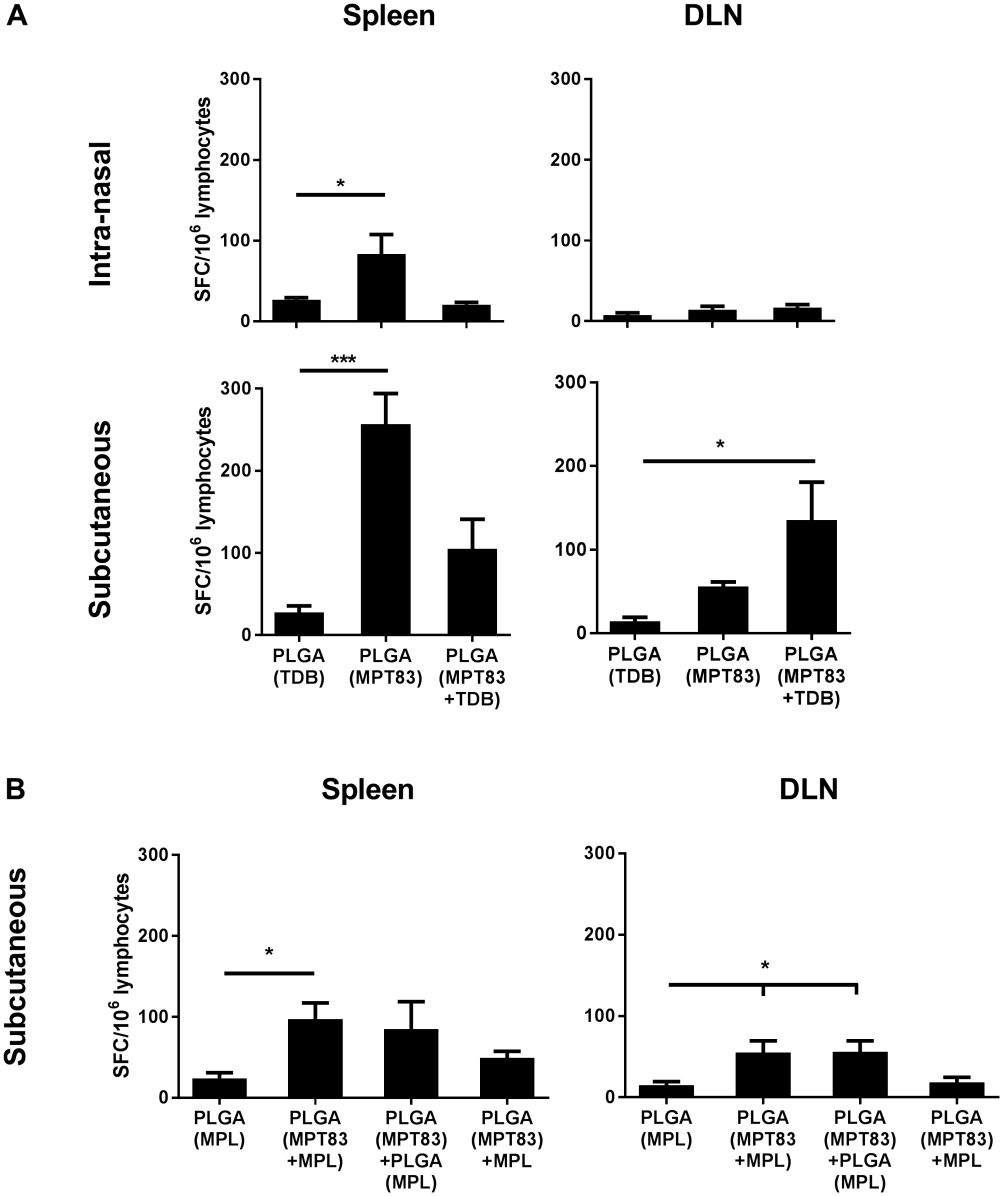
Antigen-specific IFNγ-secreting cells were induced systemically following immunisation with PLGA particulate vaccines. C57BL/6 mice were immunised with 1 mg PLGA vaccine either i.n or s.c as indicated, three times at two-weekly intervals. At four weeks after the last vaccination, lymphocytes of vaccinated mice were stimulated ex vivo with MPT83 (10 μg/ml). The numbers of MPT83-specific IFNγ-secreting cells/10^6^ lymphocytes in the spleen or DLN of mice immunised with PLGA vaccines adjuvanted with (A) TDB or (B) MPL were enumerated by ELISPOT after 20 hr antigen stimulation. The data are the means ± SEM (n = 3) and are representative of two independent experiments. Statistical significance was calculated by ANOVA with Bonferroni post-hoc comparison to adjuvant only control (*p<0.05, ***p<0.001).

To assess the immunogenicity of PLGA particulate vaccines, C57BL/6 mice were immunised by i.n or s.c routes, and four weeks after the last vaccination IFNγ-secreting T-lymphocyte responses were assessed systemically in the spleen and DLN. It was verified that mice left unimmunised or vaccinated with PLGA alone did not generate immune responses to MPT83. Mice immunised i.n with PLGA(MPT83) had a small, but significant IFNγ response in the spleen to MPT83, but not in the MLN. No response was seen after i.n immunisation with PLGA(MPT83+TDB) (Fig 1A). Mice immunised s.c with PLGA(MPT83) induced a larger and significantly increased population of MPT83-specific IFNγ-secreting cells in the spleen, with a similar trend observed for mice immunised with PLGA(MPT83+TDB) (Fig 1A). In addition, mice immunised s.c with PLGA(MPT83+TDB) had a significantly higher proportion of antigen-specific IFNγ-producing cells in the ILN (Fig 1A). The immunogenicity of PLGA(MPT83) in combination with MPL encapsulated within the same particle, in a separate PLGA particle, or external to the particle in soluble form, were also compared after peripheral immunisation. Mice immunised with PLGA(MPT83+MPL) had a small, but significant IFNγ response to MPT83 in the spleen (Fig 1B). Both PLGA(MPT83+MPL) and PLGA(MPT83)+PLGA(MPL) induced IFNγ responses in the ILN of immunised mice (Fig 1B).

### Polyfunctional CD4^+^ T-lymphocytes were induced by mucosal or peripheral immunisation

**Fig 2:**
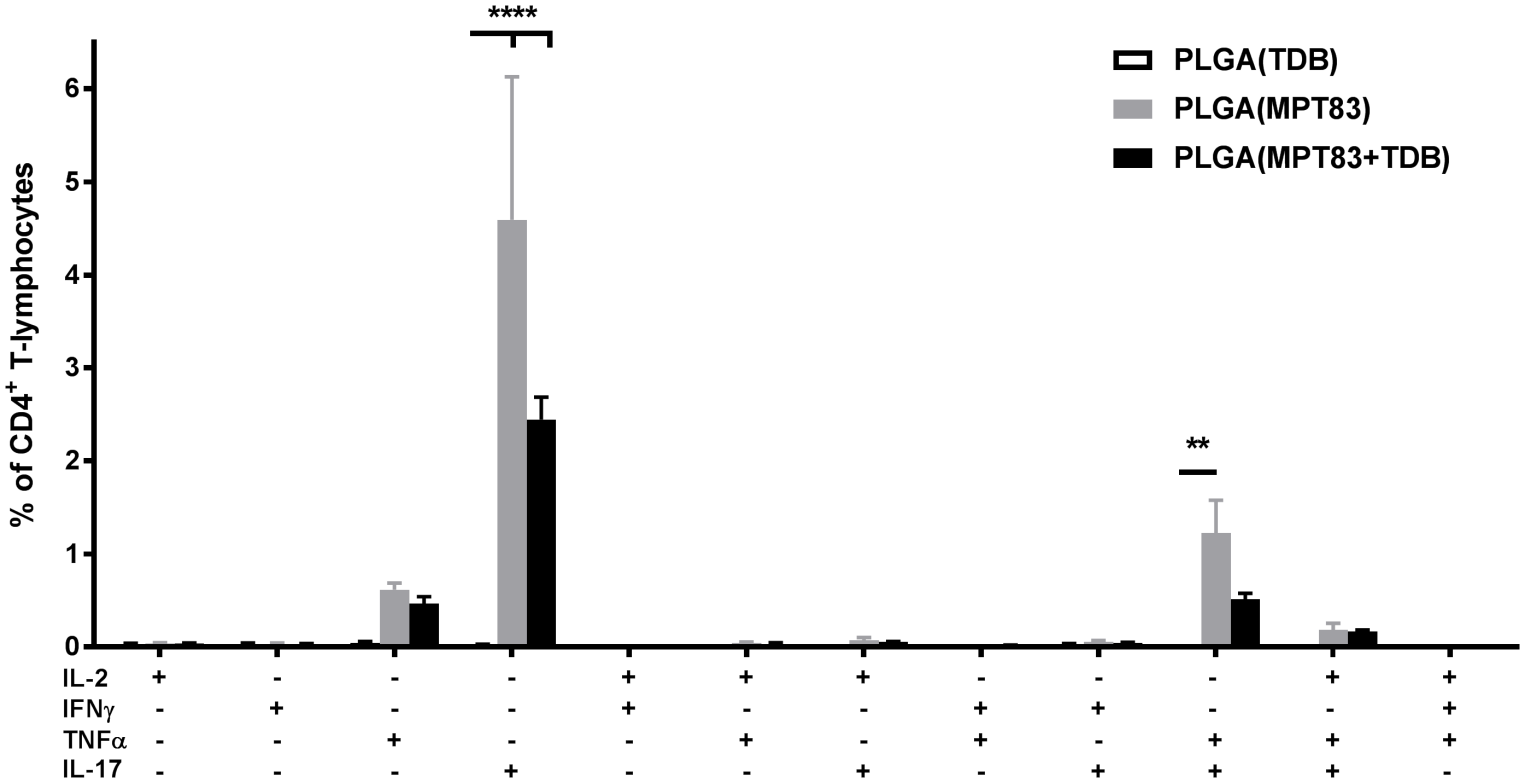
Mucosal delivery of PLGA vaccines with TDB induces antigen-specific cytokine responses locally in the lungs. Frequency of cytokine-producing CD4^+^ T-lymphocytes were assessed at four weeks following final i.n immunisation. Antigen-specific cells were detected by intra-cellular immunostaining and flow cytometry after recall with MPT83 (10 μg/ml) for 12 hr followed by addition of Brefeldin A and further incubation for 4 hr. Data are the means ± SEM (n = 3) and are representative of two independent experiments. Statistically significant differences were determined by ANOVA with post-hoc Bonferroni comparison to adjuvant only control (**p<0.01, ****p<0.0001).

**Fig 3:**
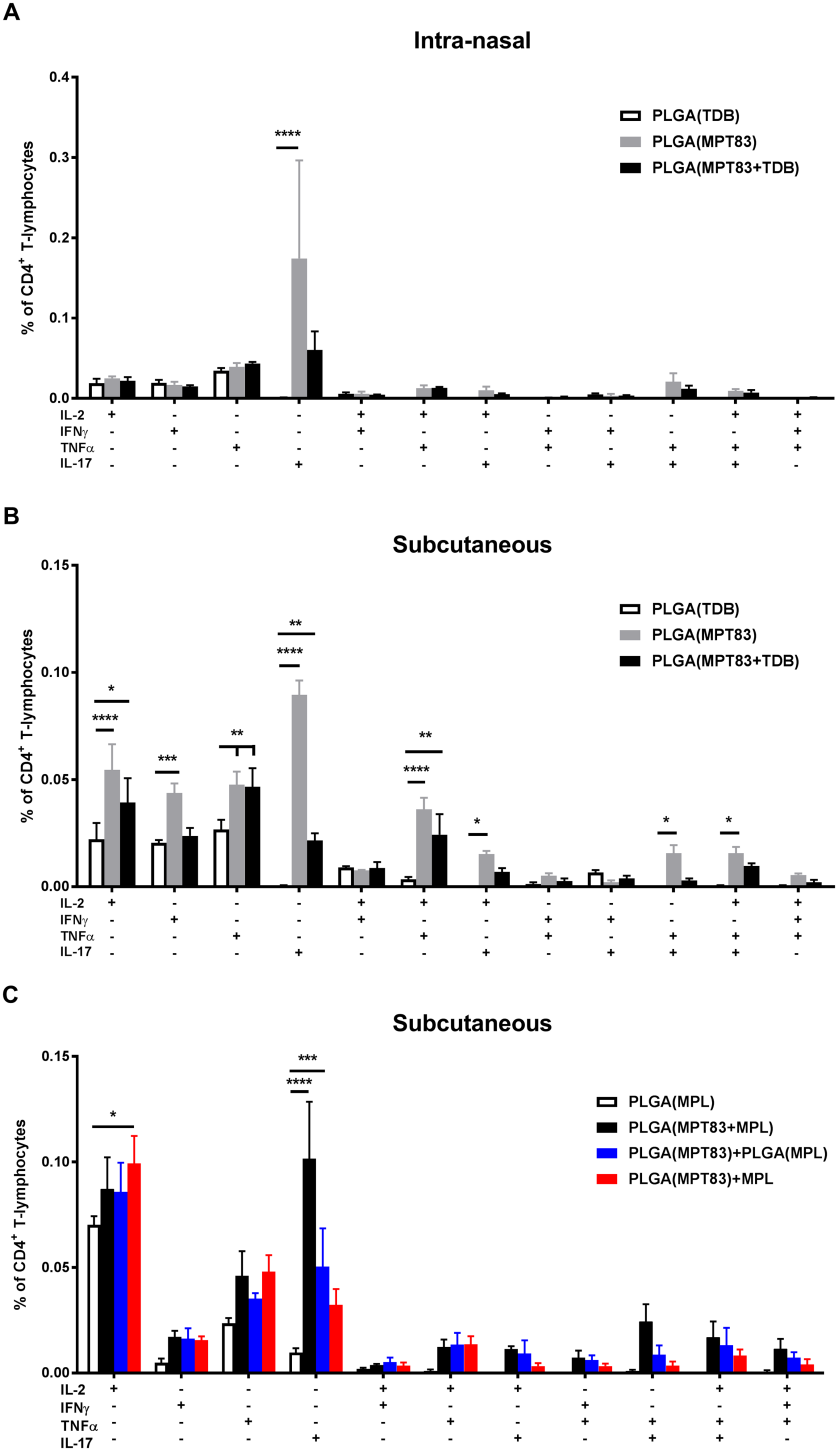
PLGA vaccination induces systemic antigen-specific cytokine responses. Frequency of cytokine-producing CD4^+^ T-lymphocytes in the spleens of mice immunised (A) i.n and (B) s.c with TDB adjuvanted vaccines, and (C) s.c with MPL adjuvanted vaccines, as assessed at four weeks following final immunisation. Antigen-specific cells were detected by intra-cellular immunostaining and flow cytometry after recall with MPT83 (10 μg/ml) for 12 hr followed by addition of Brefeldin A and further incubation for 4 hr. Data are the means ± SEM (n = 3) and are representative of two independent experiments. Statistically significant differences were determined by ANOVA with post-hoc Bonferroni comparison to adjuvant only control (*p<0.05, **p<0.01, ***p<0.001, ****p<0.0001).

To assess the capacity of antigen-specific T-lymphocytes to secrete multiple cytokines, lymphocytes from the lungs and spleen of immunised mice were recalled to MPT83 and cytokine production measured by intra-cellular immunostaining and flow cytometry. In the lungs, PLGA(MPT83) or PLGA(MPT83+TDB) i.n immunised mice had significantly increased populations of IL-17^+^ and IL-17^+^TNFα^+^ CD4^+^ T-lymphocytes (Fig 2). A small population of IL-17^+^ CD4^+^ T-lymphocytes were present in the spleen of i.n immunised mice (Fig 3A). Subcutaneous delivery of PLGA(MPT83) or PLGA(MPT83+TDB) induced a more varied Th1/Th17 phenotype of cytokine responses in the spleen and these were of a lower magnitude (Fig 3B). Cytokine responses were generally strongest in mice immunised with PLGA(MPT83) only. In comparison, after peripheral immunisation with PLGA vaccines adjuvanted with MPL, small populations of cytokine producing cells were observed in the spleen of all groups where MPT83 was included in the vaccine. These were predominantly IL-17^+^ CD4^+^ T-lymphocytes, with the highest responses seen in mice immunised with PLGA(MPT83+MPL) (Fig 3C). IFNγ, IL-2 and TNFα release by MPT83-specific CD8^+^ T-lymphocytes generated by i.n or s.c vaccination were also assessed, but responses were minimal (S1 Supporting Information). Therefore PLGA and antigen, with or without adjuvant, had an adjuvant effect and stimulated predominantly IL-17 responses.

### Lymphocyte proliferation induced by PLGA particulate vaccination

**Fig 4:**
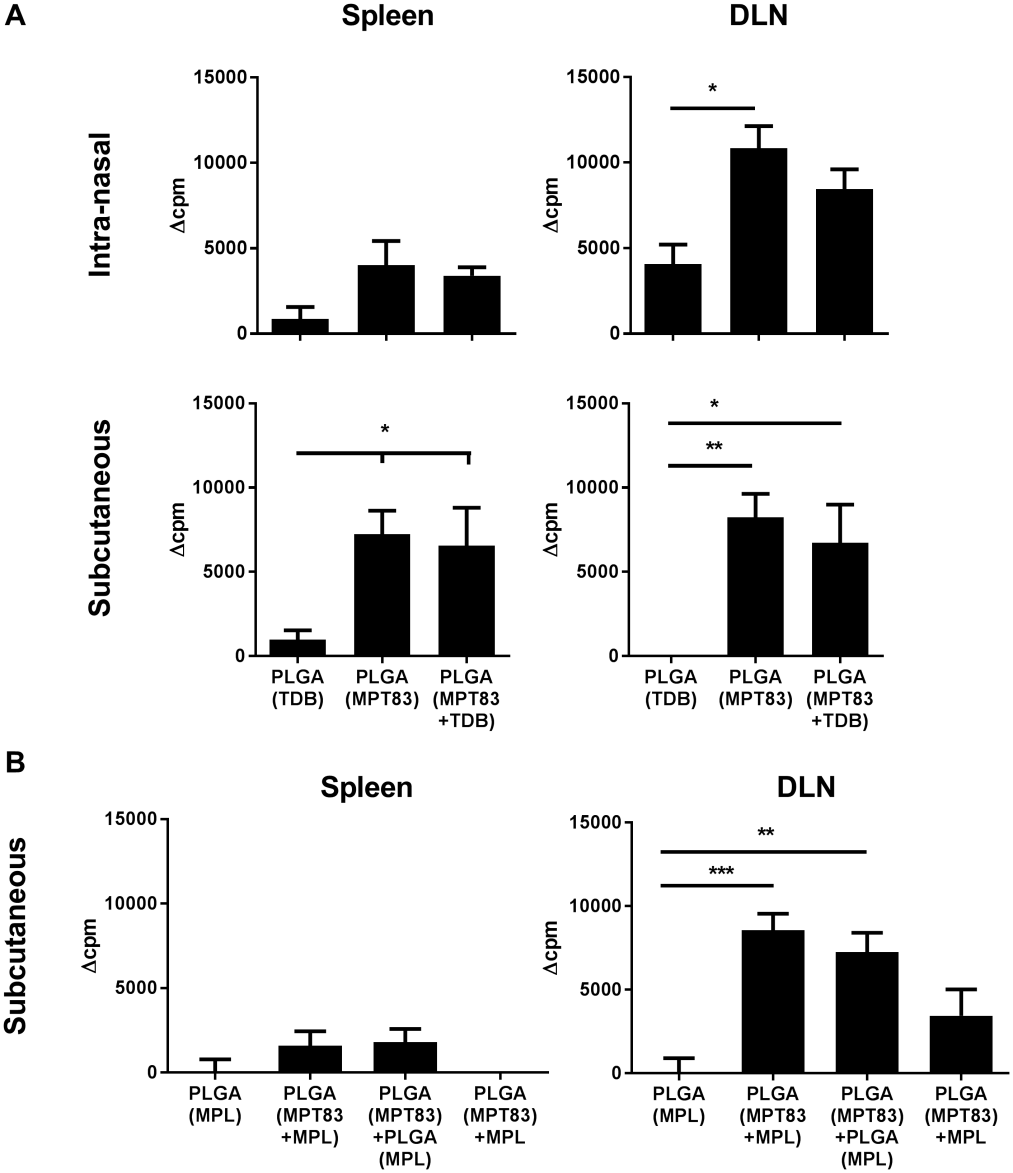
Systemic proliferative antigen-specific lymphocytes were induced following vaccination with PLGA particulate vaccines. C57BL/6 mice were immunised three times at two-weekly intervals with 1 mg PLGA vaccine, either i.n or s.c as indicated, adjuvanted with (A) TDB or (B) MPL. Leukocytes from the spleens and LN of vaccinated mice were stimulated with MPT83 (10 μg/ml) ex vivo four weeks after last vaccination. Proliferation of antigen-specific cells was determined by ^3^H-thymidine incorporation assay, measured by delta counts per minute (Δcpm; test minus the mean of negative/adjuvant only control) for immunised mice. The data are the means ± SEM (n = 3). Statistical significance was calculated by ANOVA with Bonferroni post-hoc comparison to adjuvant only control (*p<0.05, **p<0.01, ***p<0.001).

To assess further antigen-specific lymphocyte responses, leukocytes from LNs and spleens of immunised mice were stimulated with MPT83 and proliferation assessed by ^3^H-thymidine incorporation assay. Significant populations of proliferating antigen-specific cells were detected in the MLN of mice vaccinated i.n with either PLGA(MPT83) or PLGA(MPT83+TDB) (Fig 4A). An increased level of T-cell proliferation was detected in the spleens of mice immunised s.c, and a similar response was seen in the ILN (Fig 4A). In mice immunised s.c with MPL adjuvanted PLGA vaccines, the greatest degree of proliferation was seen in the ILN after PLGA(MPT83+MPL) vaccination, and this was also significantly increased in mice immunised with PLGA(MPT83)+PLGA(MPL) (Fig 4B). Therefore PLGA-based vaccination induces significant T-lymphocyte activation, but with minimal IFN-γ producing cells (Figs 2 and 3).

### IgG responses to mucosal or peripheral immunisation with PLGA particles

**Fig 5:**
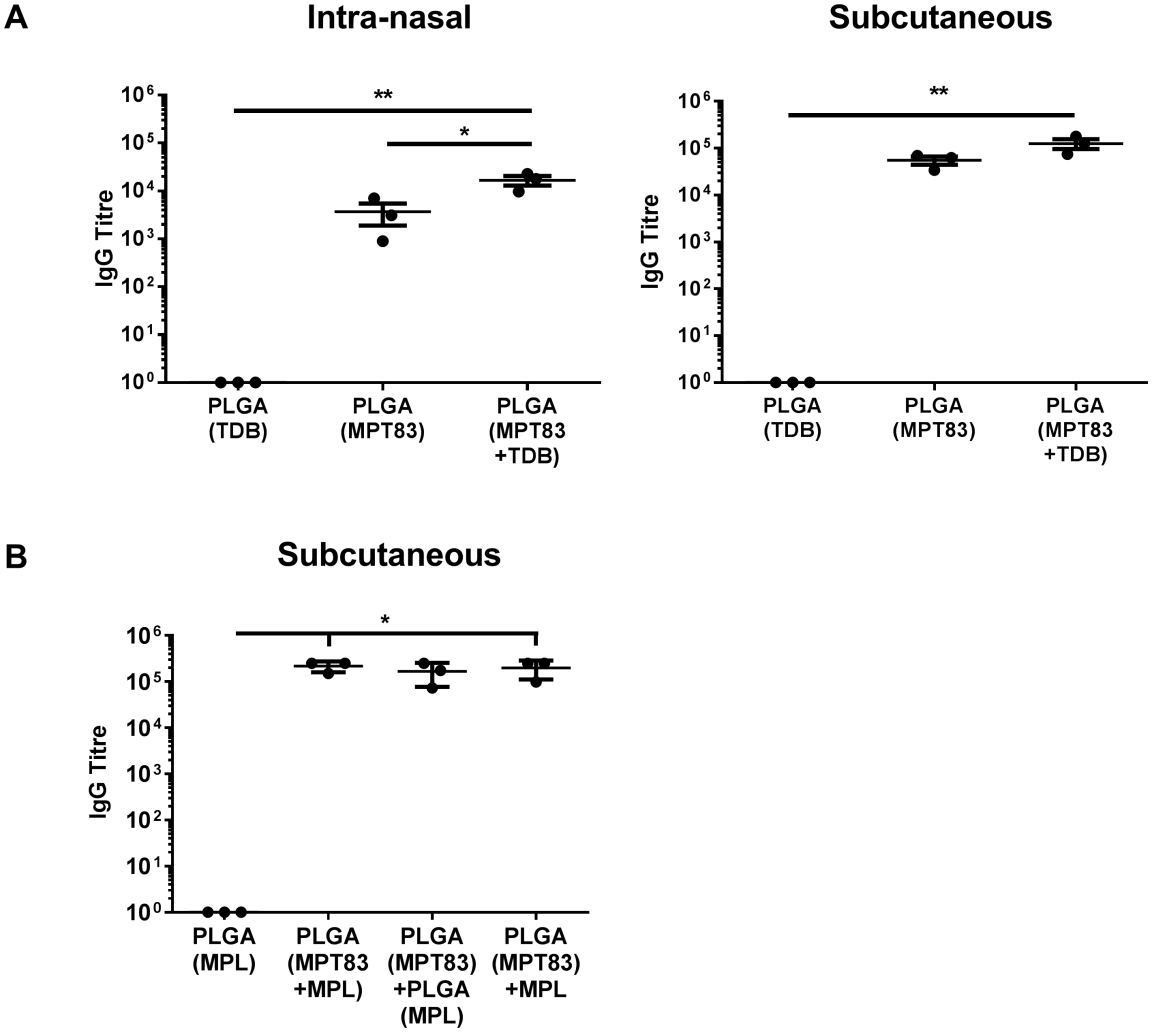
PLGA vaccination elicited potent systemic anti-MPT83 IgG responses. C57BL/6 mice (n = 3) were immunised with 1 mg PLGA vaccine, three times at two-weekly intervals. 28 days following final immunisation anti-MPT83 IgG was detected by ELISA in the serum of mice immunised (A) i.n or s.c with TDB adjuvanted vaccine, or (B) s.c with MPL adjuvanted vaccine. Titre was determined as the highest dilution giving an absorbance greater than the mean absorbance of a 1:100 dilution of adjuvant only immunised mouse sera. The data are the means ± SEM and are representative of two independent experiments. Statistical significance was calculated by ANOVA with Bonferroni post-hoc comparison (*p<0.05, **p<0.01).

Mice were immunised as above, the serum collected four weeks after the last immunisation, and MPT83-specific IgG titres measured by ELISA. Immunisation with PLGA(MPT83) or PLGA(MPT83+TDB), by either route, induced significant systemic antigen-specific IgG titres (Fig 5A). The antibody response was most potent when TDB was incorporated with MPT83 in the PLGA particle, and when given by the s.c route (Fig 5A). In studies utilising MPL adjuvanted PLGA vaccines, potent titres of systemic antigen-specific IgG were seen in all groups receiving MPT83 in the vaccine. Immunisation with PLGA(MPT83+MPL) provided the highest titres, however these were similar to that provided by immunisation with antigen and adjuvant in separate particles, or PLGA(MPT83)+MPL (Fig 5B). Therefore, PLGA alone with antigen acts as an antibody-promoting adjuvant and this effect was increased to an extent by the addition of an additional adjuvant.

### Protective efficacy of PLGA particulate subunit vaccination

**Fig 6:**
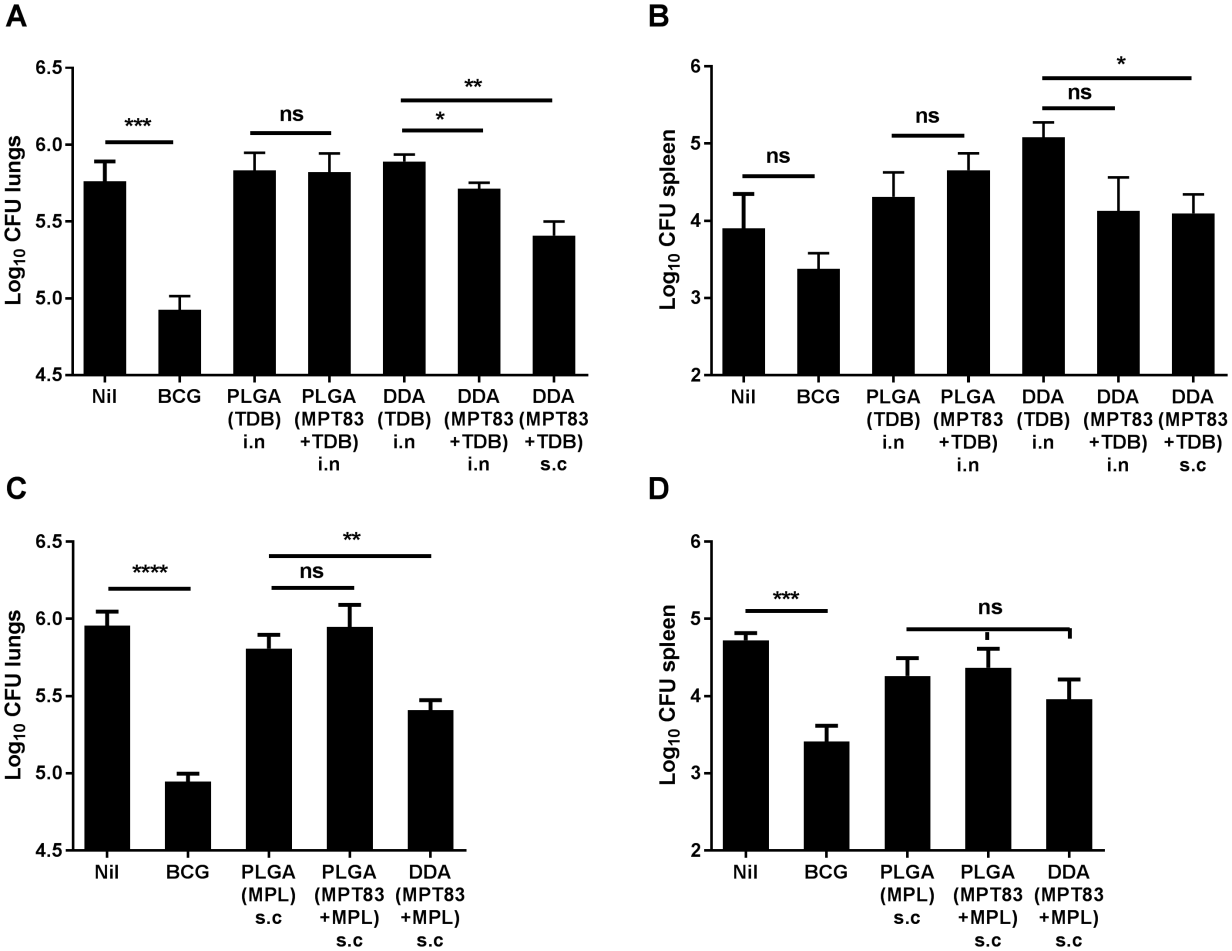
PLGA particulate vaccination failed to induce protection against *M*. *tuberculosis* infection. C57BL/6 mice were immunised three times at two-weekly intervals with PLGA particles (n = 6) or DDA liposomes (n = 3–6), containing MPT83 adjuvanted with TDB, (A) lungs and (B) spleen, or adjuvanted with MPL, (C) lungs and (D) spleen. Six weeks following the final vaccination, mice were challenged with a low-dose aerosol of *M*. *tuberculosis* H37Rv (100 CFU). Additional mice (n = 6) were immunised with 5×10^5^ CFU BCG once by s.c injection 12 weeks before challenge. After 4 weeks the bacterial loads were enumerated in mice immunised either i.n or s.c as indicated. The data are the means ± SEM. Statistical significance was determined by Student’s *t*-test, comparing the vaccinated group to the relevant unimmunised/adjuvant only control (*p<0.05, **p<0.01, ***p<0.001, ****p<0.0001).

We had previously shown that s.c DDA(MPT83+MPL) induced protective immunity in the lungs against aerosol *M*. *tuberculosis* infection [21]. Immune responses to peripherally delivered DDA(MPT83+TDB) and DDA(MPT83+MPL) were also assessed, and unlike PLGA particulate-based vaccines, these liposome formulations were highly immunogenic, inducing potent Th1, Th17 and CD8^+^ T-lymphocyte responses as well as antibody responses (S1–S3 Figs). PLGA particulate vaccines were therefore compared to DDA-liposome-based vaccines containing MPT83 and TDB/MPL in protection studies. Mice were rested for six weeks after the final immunisation, then infected by aerosol with *M*. *tuberculosis* H37Rv (100 CFU). The bacterial loads in the lungs and spleen were enumerated four weeks after challenge. No protective effect was seen in the lungs or spleen after i.n PLGA(MPT83+TDB) (Fig 6A and 6B). Similarly, no protective effect was seen in the lungs or spleen after s.c PLGA(MPT83+MPL) vaccination (Fig 6C and 6D). In contrast, when MPT83 and TDB/MPL was formulated into DDA-based liposomes, these stimulated protection against *M*. *tuberculosis*, primarily in the lungs (Fig 6A and 6C). PLGA particles or DDA liposomes alone did not cause exacerbation of infection. The bacterial burden in the spleens of unimmunised mice in Fig 6B was lower than usually observed, and as a result BCG immunisation did not provide significant protection in the spleen. The bacterial load in the spleens of DDA(TDB) immunised control mice in Fig 6B is typical of the bacterial burden observed in unimmunised mice, and BCG immunisation provided protection in the spleen as well as lungs (Fig 6A, 6C and 6D).

## Discussion

There is an urgent need for the development of more effective, safe and easily administrable vaccines for TB. Protein-based subunit vaccines have the potential to fulfil these requirements, but require immunostimulatory adjuvants to activate dendritic cells and stimulate a response that will lead to long term memory. There is also interest in exploring pulmonary vaccine delivery for TB. This approach has been utilised successfully in clinical trials for other respiratory pathogens, such as measles [39] and influenza, including when the vaccine was self-administered by study participants [40]. This study compared the effects of mucosal or peripheral immunisation with *M*. *tuberculosis* lipoprotein, MPT83, a known protective antigen [26], and adjuvants targeting PRRs contained within slow release PLGA particles, and their protective efficacy was contrasted with DDA-based cationic liposomal delivery of the same antigen and adjuvants.

The use of micro- or nanoparticles for vaccine delivery is being explored for a number of reasons. Encapsulation of antigen and adjuvant in microparticles/liposomes may not only provide protein to APCs in the context of immunostimulatory signals without the systemic adverse effects caused by soluble immunopotentiators [41], but also has the advantage of protecting vaccine components from the hostile external environment. Additionally, they may overcome problems related to solubility for the delivery of lipid-based vaccine components [18]. Microparticles produced from certain polymers, such as PLGA, provide the capacity to formulate vaccines designed for slow release of antigen and adjuvant, prolonging innate immune stimulation and potentially leading to improved adaptive immune responses [10]. PLGA is already approved for clinical use, and so clinical development of particulate vaccines based on this polymer is feasible [42]. There is also potential to produce powdered PLGA particles using spray or freeze drying in a readily scalable and reproducible process [17, 36].

There is evidence that delivery of antigen in a particulate form provides greater strength and duration of T-lymphocyte responses compared to delivery of soluble antigen [43]. It has also been suggested that the slow release of antigen achieved by PLGA encapsulation, in contrast to liposomes, provides superior humoral and long-term CD8^+^ T-lymphocyte responses [10, 11]. In our studies, however, PLGA particles were less effective at stimulating strong Th1/Th17 CD4^+^ and CD8^+^ T-lymphocyte systemic responses (Figs 1 and 3), whereas DDA liposome formulations were potently immunogenic (S1–S3 Figs), consistent with other previous studies [44]. Notably the addition of the adjuvants TDB and MPL to the PLGA particles did not increase T-cell responses, however when pulmonary delivery of PLGA particles was utilised, improved responses were seen locally in the lungs, particularly IL-17-secreting CD4^+^ T-lymphocyte responses (Fig 2). Despite these Th17 responses, however, no PLGA vaccine tested in this study provided protection from *M*. *tuberculosis* in the murine aerosol challenge model (Fig 6).

Pulmonary or mucosal vaccination in general appears to favour the induction of a Th17 T-lymphocyte response, regardless of the adjuvant used [45], however the role of IL-17 in vaccine-induced protection against *M*. *tuberculosis* is controversial [46, 47]. IL-17 induces the expression of pro-inflammatory cytokines and chemokines, such as G-CSF, IL-6 and IL-8, promoting recruitment of neutrophils and granuloma formation [48, 49], as well as the expression of other chemokines, including CXCL10 that recruits IFNγ producing cells to the site of infection [50]. In some contexts, IL-17 has been reported as critical for enhanced protection against *M*. *tuberculosis* [51]. A recent study assessed protection from *M*. *tuberculosis* challenge in DBA/2 mice, in which parenterally administered BCG vaccination is not effective. Notably, intra-nasal BCG vaccination was protective in this model, and was dependent on the anti-*M*. *tuberculosis* Th17 response [52]. However, a disproportionate IL-17 response may lead to excessive neutrophil recruitment, resulting in immunopathological injury [53, 54]. Our study is strongly suggestive that a Th17 response alone to a vaccine antigen is unlikely to deliver protective efficacy.

A striking feature of the PLGA vaccines were the high titres of circulating antigen-specific IgG responses achieved, which were improved by the addition of adjuvant, either TDB or MPL, and were greatest after s.c delivery (Fig 5). The data are therefore strongly suggestive that PLGA itself as a vaccine carrier has adjuvant activity and tends to bias the immune response towards antibody production which was not altered by the addition of TDB or MPL. This may be influenced by the properties of PLGA to release antigen slowly, which may drive the selection of high affinity B-cells and therefore antibody responses [55], as well as the size of the particles, with vaccine particles in the μm size range reported to preferentially drive Th2 responses [56]. Notably, regardless of the route of administration, the PLGA-based vaccines stimulated antigen-specific proliferative responses, confirming T-cell activation (Fig 4). It therefore appears that PLGA microparticles skew the immune response towards the development of Th2-like CD4^+^ T-lymphocyte responses, resulting in predominantly antibody responses to the vaccine antigen. To achieve this the adjuvant did not need to be encapsulated within the same particle as MPT83, but could be encapsulated in a separate particle and co-delivered, or delivered with PLGA(MPT83) in a soluble form (Fig 5B). The ability to deliver antigen and adjuvant in separate particles offers flexibility in vaccine design, whereby a generic adjuvant containing particle may be produced and then combined with a particle containing antigen from the pathogen of choice [11]. However, despite the induction of a strong IgG response against the vaccine antigen, this was not sufficient for protection. Recently, antibodies against capsular polysaccharides have been reconsidered as protective against *M*. *tuberculosis* in mice [57]. Therefore an alternative approach may be to consider vaccinating with a glycosylated protein antigen, or with protein conjugated to surface lipids or carbohydrates from *M*. *tuberculosis*.

Together, our study is strongly suggestive that a vaccine-induced Th17 or antibody response is not sufficient for protection against *M*. *tuberculosis*. The data are consistent with a previous study that examined PLGA particles containing rAg85B and TDB, formulated as a spray-dried powder for pulmonary delivery to guinea pigs [58]. As a homologous vaccine, no significant protective effect was generated after aerosol challenge with *M*. *tuberculosis*, although the histopathology scores of infected lungs were improved. When utilised as a boosting immunisation following s.c BCG, however, a reduced bacterial burden was achieved in the lungs and spleen [58]. It therefore appears unlikely that homologous PLGA-based vaccination will be a suitable strategy for translation to clinical trials for the prevention of TB, but there is potential for mucosal PLGA-based vaccines as booster immunisations. There is also potential to incorporate several antigens and adjuvants within these particles, targeting combinations of PRRs to induce multiple signalling pathways, such as MyD88 and Syk/Card9, in order to improve Th1 responses [11]. Recent studies have utilised two ligands for TLR4 and TLR7, in separate nanoparticles to the antigen [11], or ligands against TLR3 and TLR9 within the same particle as antigen [59], to increase synergistically neutralising antibodies, as well as CD4^+^ and CD8^+^ T-lymphocyte responses. In contrast, DDA-formulated peripherally delivered vaccines containing the same antigen and adjuvants provided potent Th1 and Th17 responses to the vaccine antigen (S1 and S2 Figs), and achieved significant protection in the lungs of *M*. *tuberculosis* challenged mice (Fig 6), offering clear potential for future applications.

In conclusion, PLGA as a vaccine carrier is a potent inducer of antibody responses. While antigen containing PLGA particulate vaccines did activate T cells, the capacity to induce Th1/Th17 responses was limited, although local IL-17-producing T-cell responses were enhanced by mucosal delivery. The immune response induced by these vaccines, regardless of the route of delivery, were not protective against *M*. *tuberculosis* in mice. In contrast, DDA liposome-based vaccines were highly immunostimulatory and provided a significant degree of protection in a murine model of *M*. *tuberculosis* aerosol challenge. This study demonstrates that a careful selection of delivery system, adjuvant and route of vaccination are essential considerations in the design of novel vaccines for the prevention of TB. The combination of particulate formulation and pulmonary delivery offer potential as an easily administrable vaccination strategy, with potential for application to other infectious diseases where protection is primarily mediated by humoral immunity.

## Supporting information

S1 Fig Peripheral DDA(MPT83+TDB) vaccination induces strong systemic antigen-specific cytokine responses. C57BL/6 mice (n = 3) were left unimmunised (open bars) or injected s.c with DDA(MPT83+TDB) liposomes (closed bars) three times at two-weekly intervals. Proportion of cytokine-producing (A) CD4^+^ and (B) CD8^+^ T-lymphocytes in the spleens of immunised mice were assessed at four weeks following final immunisation. Antigen-specific cells were detected by intra-cellular immunostaining and flow cytometry after recall with MPT83 (10 μg/ml). Data are the means ± SEM (n = 3) and are representative of two independent experiments. Statistically significant differences were determined by ANOVA with post-hoc Bonferroni comparison (*p<0.05, ****p<0.0001).
(TIF)

S2 Fig Peripheral DDA(MPT83+MPL) vaccination induces systemic antigen-specific cytokine responses. C57BL/6 mice (n = 2–4) were left unimmunised (open bars) or injected s.c with DDA(MPT83+MPL) liposomes (closed bars) three times at two-weekly intervals. Proportion of cytokine-producing (A) CD4^+^ and (B) CD8^+^ T-lymphocytes in the spleens of immunised mice were assessed at 4 weeks following final immunisation. Antigen-specific cells were detected by intra-cellular immunostaining and flow cytometry after recall with MPT83 (10 μg/ml). Data are the means ± SEM and are representative of two independent experiments. Statistically significant differences were determined by ANOVA with post-hoc Bonferroni comparison to unimmunised controls (*p<0.05, **p<0.01, ***p<0.001, ****p<0.0001).
(TIF)

S3 Fig Subcutaneous DDA liposome-based vaccination elicited potent systemic anti-MPT83 IgG responses. C57BL/6 mice (n = 2–4) were left unimmunised or were injected s.c with (A) DDA(MPT83+TDB) or (B) DDA(MPT83+MPL) liposomes, three times at two-weekly intervals. Mice were euthanised four weeks following final immunisation and anti-MPT83 IgG detected by ELISA in the sera. Titre was determined as the highest dilution giving an absorbance greater than the mean absorbance of a 1:100 dilution of unimmunised mouse sera. The data are the means ± SEM and are representative of two experiments.
(TIF)

S1 Supporting Information Data sets used in analysis of vaccine efficacy. (XLSX)
