# Supplementary figures and images for: PLGA particulate subunit tuberculosis vaccines promote humoral and Th17 responses but do not enhance control of Mycobacterium tuberculosis infection
Source: PLoS One. 2018 Mar 19;13(3):e0194620. doi: 10.1371/journal.pone.0194620 (PMC5858788; doi:10.1371/journal.pone.0194620)

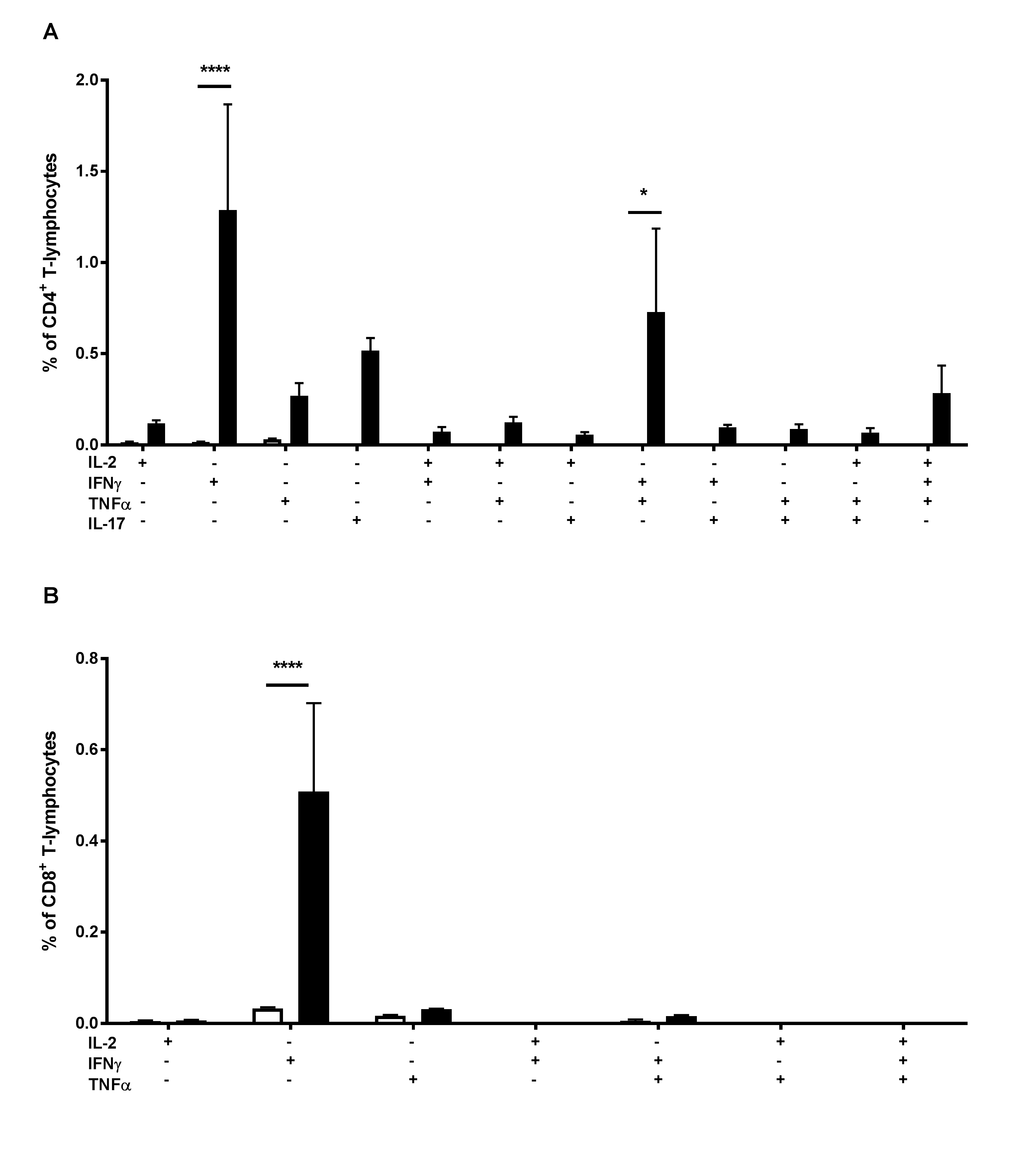

Supplement: S1 Fig — C57BL/6 mice (n = 3) were left unimmunised (open bars) or injected s.c with DDA(MPT83+TDB) liposomes (closed bars) three times at two-weekly intervals. Proportion of cytokine-producing (A) CD4+ and (B) CD8+ T-lymphocytes in the spleens of immunised mice were assessed at four weeks following final immunisation. Antigen-specific cells were detected by intra-cellular immunostaining and flow cytometry after recall with MPT83 (10 μg/ml). Data are the means ± SEM (n = 3) and are representative of two independent experiments. Statistically significant differences were determined by ANOVA with post-hoc Bonferroni comparison (*p<0.05, ****p<0.0001). (TIF) [file pone.0194620.s001.tif]

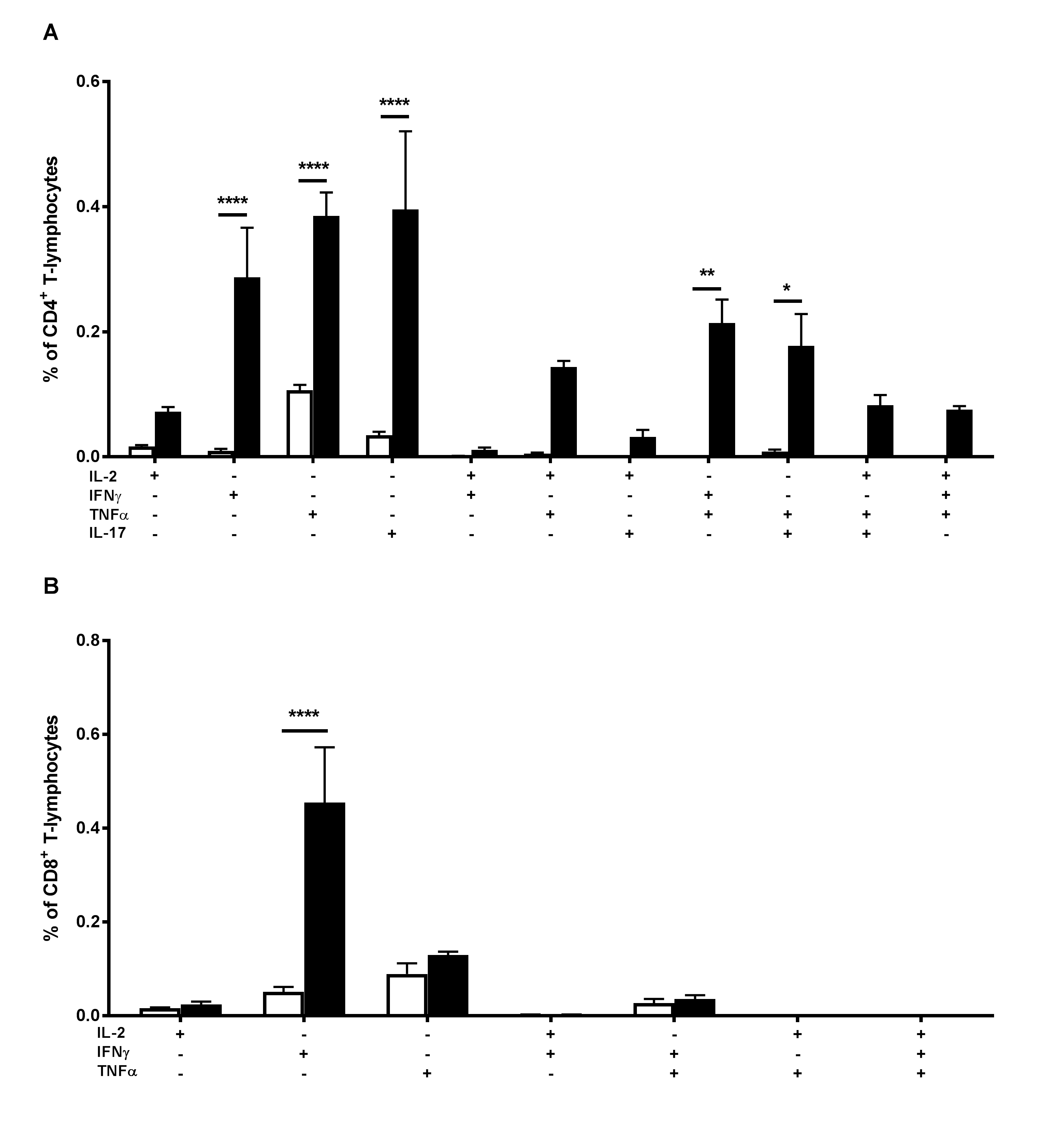

Supplement: S2 Fig — C57BL/6 mice (n = 2–4) were left unimmunised (open bars) or injected s.c with DDA(MPT83+MPL) liposomes (closed bars) three times at two-weekly intervals. Proportion of cytokine-producing (A) CD4+ and (B) CD8+ T-lymphocytes in the spleens of immunised mice were assessed at 4 weeks following final immunisation. Antigen-specific cells were detected by intra-cellular immunostaining and flow cytometry after recall with MPT83 (10 μg/ml). Data are the means ± SEM and are representative of two independent experiments. Statistically significant differences were determined by ANOVA with post-hoc Bonferroni comparison to unimmunised controls (*p<0.05, **p<0.01, ***p<0.001, ****p<0.0001). (TIF) [file pone.0194620.s002.tif]

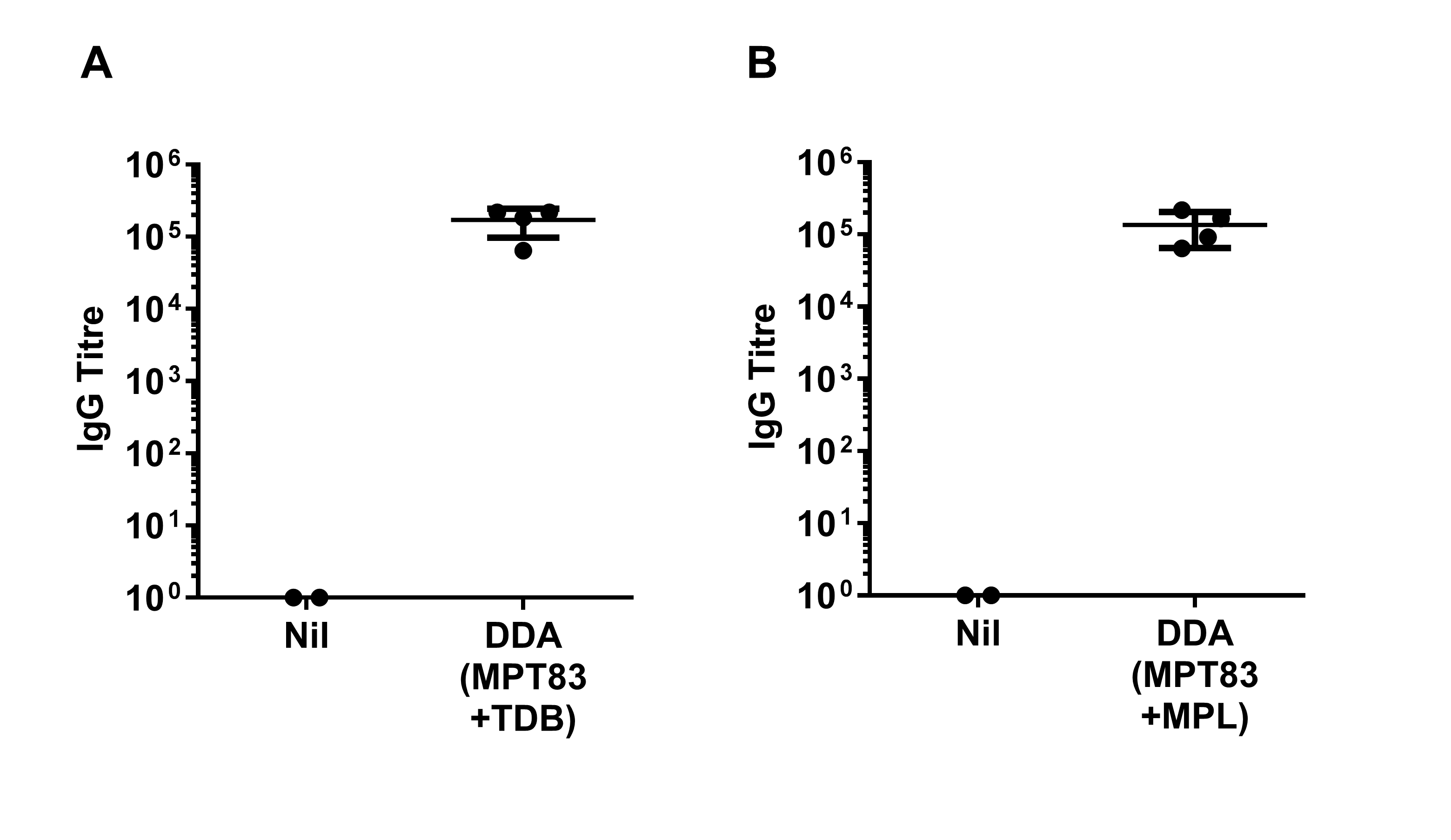

Supplement: S3 Fig — C57BL/6 mice (n = 2–4) were left unimmunised or were injected s.c with (A) DDA(MPT83+TDB) or (B) DDA(MPT83+MPL) liposomes, three times at two-weekly intervals. Mice were euthanised four weeks following final immunisation and anti-MPT83 IgG detected by ELISA in the sera. Titre was determined as the highest dilution giving an absorbance greater than the mean absorbance of a 1:100 dilution of unimmunised mouse sera. The data are the means ± SEM and are representative of two experiments. (TIF) [file pone.0194620.s003.tif]
